# The risk of carotid plaque instability in patients with metabolic syndrome is higher in women with hypertriglyceridemia

**DOI:** 10.1186/s12933-021-01277-8

**Published:** 2021-05-06

**Authors:** Francesca Servadei, Lucia Anemona, Marina Cardellini, Manuel Scimeca, Manuela Montanaro, Valentina Rovella, Francesca Di Daniele, Erica Giacobbi, Iacopo Maria Legramante, Annalisa Noce, Rita Bonfiglio, Patrizia Borboni, Nicola Di Daniele, Arnaldo Ippoliti, Massimo Federici, Alessandro Mauriello

**Affiliations:** 1 Anatomic Pathology, Department of Experimental Medicine, University of Rome Tor Vergata, Via Montpellier 1, Rome, RM 00133 Italy; 2 Department of Systems Medicine, University of Rome Tor Vergata, Rome, Italy; 3 San Raffaele University, Via di Val Cannuta 247, 00166 Rome, Italy; 4 Saint Camillus International University of Health Sciences, Via di Sant’Alessandro, 8, 00131 Rome, Italy; 5 UOC of Internal Medicine, Center of Hypertension, University of Rome Tor Vergata, Rome, Italy; 6 PhD School of Applied Medical, Surgical Sciences, University of Rome Tor Vergata, Rome, Italy; 7 Fondazione Umberto Veronesi (FUV), Piazza Velasca 5, MI 20122 Milano, Italy; 8 Vascular Surgery, Department of Biomedicine and Prevention, University of Rome Tor Vergata, Rome, Italy; 9 Tor Vergata Oncoscience Research (TOR), University of Rome “Tor Vergata“, Rome, Italy

**Keywords:** Metabolic syndrome, Carotid, Histology, Hypertriglyceridemia, Post‐menopause

## Abstract

**Background:**

Metabolic syndrome certainly favors growth of carotid plaque; however, it is uncertain if it determines plaque destabilization. Furthermore, it is likely that only some components of metabolic syndrome are associated with increased risk of plaque destabilization. Therefore, we evaluated the effect of different elements of metabolic syndrome, individually and in association, on carotid plaques destabilization.

**Methods:**

A total of 186 carotid endarterectomies from symptomatic and asymptomatic patients were histologically analysed and correlated with major cardiovascular risk factors.

**Results:**

Metabolic syndrome, regardless of the cluster of its components, is not associated with a significant increase in risk of plaque destabilization, rather with the presence of stable plaques. The incidence of unstable plaques in patients with metabolic syndrome is quite low (43.9 %), when compared with that seen in the presence of some risk factors, but significantly increases in the subgroup of female patients with hypertriglyceridemia, showing an odds ratio of 3.01 (95% CI, 0.25–36.30).

**Conclusions:**

Our data may help to identify patients with real increased risk of acute cerebrovascular diseases thus supporting the hypothesis that the control of hypertriglyceridemia should be a key point on prevention of carotid atherosclerotic plaque destabilization, especially in post-menopausal female patients.

## Background

Many studies have demonstrated that metabolic syndrome (MetS), defined as a cluster of interconnected metabolic risk factors (including abdominal obesity, elevated fasting glucose, hypertriglyceridemia, hypertension, and low High-Density Lipoprotein (HDL) cholesterol levels), increases the risk for atherosclerosis and cardiovascular disease [1–6]. In addition, the recent interest of the scientific community has focused on the possible involvement of insulin resistance as a linking factor [7–10]. Various MetS components are associated with both carotid stiffness and increased plaque volume, which are highly predictive of stroke. These evidences suggest the involvement of several biological mechanisms as a potential link between impaired vascular health and MetS, such as the release of both adipocytokines with pro-inflammatory and free fatty acids from visceral adipose tissue which, reaching the liver through the portal circulation, contribute to the onset of insulin resistance and hyperlipidemia [11].

Atherosclerosis is a chronic inflammatory disease, to whose clinical manifestations multiple cellular and systemic events contribute [12]. Two types of atherosclerotic disease are described: a stable form (with low embolic risk, constant and slow growth of the plaque over time) and an unstable form (with a high embolic risk, linked to the presence of a significant inflammation which causes the rupture of the plaque, with thrombosis and subsequent fragment’s detachment resulting in embolic complications and leading to acute clinical manifestations) [13–15]. Despite it is known that the cardiovascular risk factors certainly facilitate the development and growth of atherosclerotic plaque, it is not yet fully understood exactly which in particular favor its destabilization by accelerating plaque growth with consequent rupture and thrombosis [16–18]. It is possible to hypothesize that the main cardiovascular risk factors can play a crucial role in the destabilization process of atheromatous lesions through the modification of the histological composition of fibroatheromatous plaques. The identification of specific risk factors associated with plaque destabilization is particularly important in the prevention and treatment of cerebrovascular syndromes. According to AHA/ACC guidelines, severe carotid stenosis is the main predisposing condition for acute cerebrovascular events and it still represents the most important criterion to identify patients who need surgical treatment [19–22]. Nevertheless, whether it is necessary to perform carotid endarterectomy in all asymptomatic patients with stenosis > 70 % (even if most of them have stable plaque that is not at risk of thromboembolism), is still an open question. Indeed, it is now clear that the degree of stenosis alone is not sufficient to accurately identify patients at high risk of developing an acute cerebrovascular event. Thus, in this scenario, the identification of risk factors correlated with plaque destabilization becomes necessary to calculate the most real probability of ischemic cerebrovascular complications, stratifying patients with carotid atherosclerosis.

Previous clinical studies have consistently demonstrated a significant association between MetS and incidence of ischemic stroke [2, 6, 23]. However, the relationship between MetS and the morphological characteristics of carotid plaque instability remains uncertain. Indeed, previous studies have mainly used the imaging, in particular the measurement of carotid Intimal-Medial Thickness (IMT) to assess the risk of ischemic stroke [8, 24, 25]. This represents a valid approach to evaluate the degree of atherosclerosis, but it does not provide information on the possible destabilization of plaques, that can only be assessed by histological methods. Furthermore, it has not yet been clarified which of plaque’s individual components are associated with an increased stroke risk.

Therefore, in this study we investigate each main factor involved in MetS, in order to estimate their impact on the destabilization of the carotid plaque. Specifically, to quantify their possible synergistic effect, the relative risk of different components of metabolic syndrome, individually and in association with each other, was calculated.

## Methods

### Cases selection and histology

A total of 186 carotid plaques from symptomatic (major stroke or transient ischemic attack—TIA) and asymptomatic patients submitted to surgical carotid endarterectomy (CEA) at the University of Tor Vergata (Rome, Italy) from 2016 to date were retrospectively analysed. The sampling collection and analysis methods have been previously reported [14]. In short, samples were fixed in 10 % buffered formalin, briefly decalcified using the Surgipath Decalcifer II (LEICA, Western Road, Stratford Upon Avon, UK) in order to allow the cutting without artifacts and to preserve the recognition of perfect morphology of the plaque, cut transversely every 5 mm, embedded in paraffin, and stained with haematoxylin-eosin. Only intact carotid plaques, from patients with a complete clinical and laboratory assessment of the major cardiovascular risk factors were histologically analysed and included in the study.

**Fig. 1:**
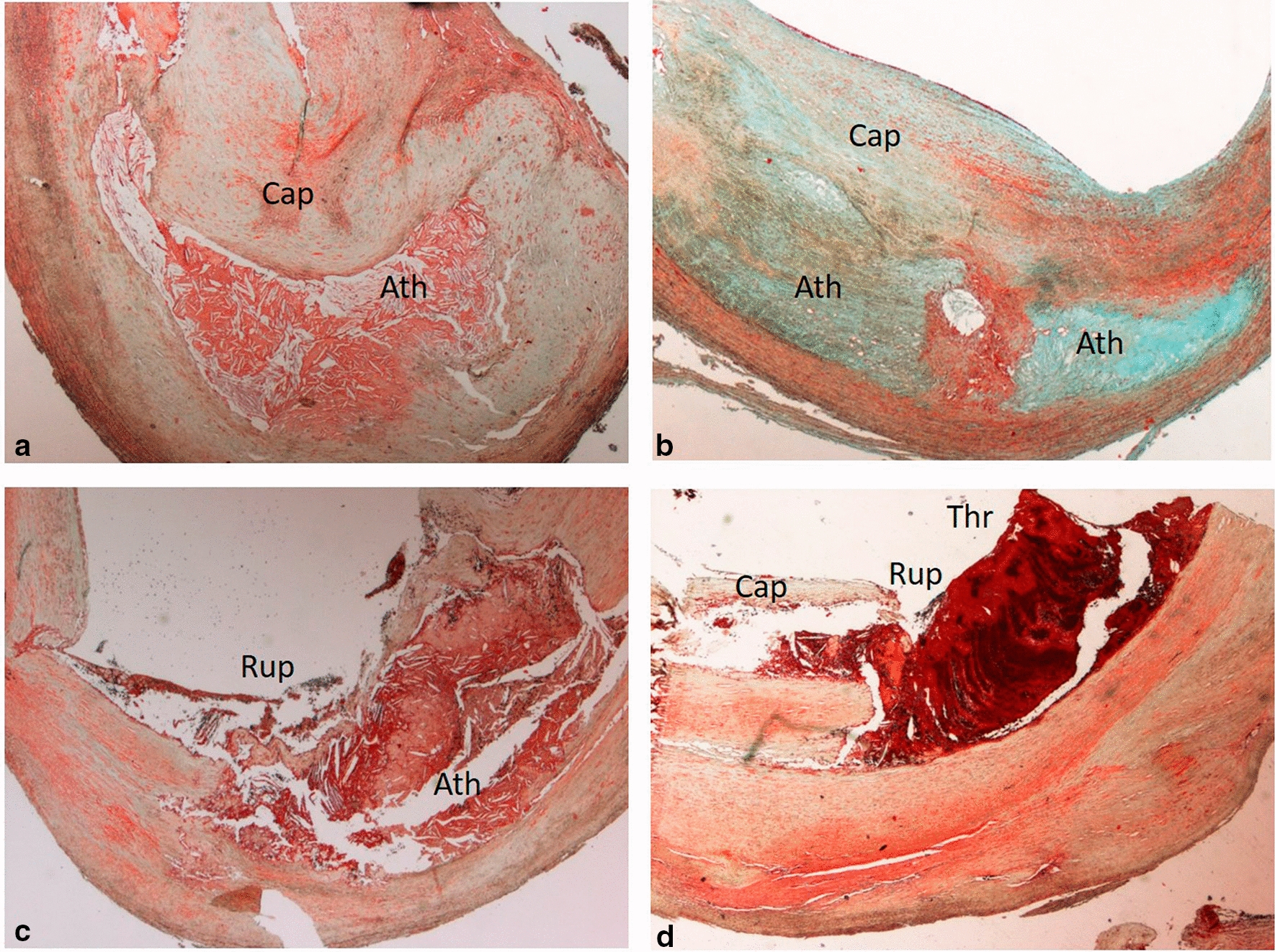
Histology of carotid plaques. **a**, **b** Stable plaque characterized by a thick fibrous cap and a large lipidic necrotic core with few inflammatory cells (Movat, 2x); **c**, **d** Unstable plaque constituted by a thrombotic plaque associated to the cap rupture (Movat, 2x). *Cap: fibrous cap; Ath: lipidic necrotic core; Thr: acute thrombus; Rup: site of cap rupture

According to the modified American Heart Association (AHA) classification, atherosclerosis plaques have been histologically distinguished into unstable and stable [26]. Unstable plaques consisted of: (a) thrombotic plaques associated with rupture or erosion of the cap; (b) healed plaque with a thrombus in organization; (c) vulnerable plaque or thin-cap fibro-atheroma (TCFA) characterized by a fibrous cap less than 165 μm thick, heavily infiltrated by macrophages CD68 positive (> 25 per high magnification field), without plaque rupture. The other plaques, classified as stable, included: (a) fibroatheromata, such as plaques with a large lipidic necrotic core and thick non-inflamed cap, (b) fibrocalcific plaques with large calcification without extensive inflammation and (c) fibrous plaques mainly constituted by fibrous tissue (Fig. 1).

Immunohistochemical analysis was performed in all plaques in order to characterize the inflammatory infiltrate using CD68 antibody anti-monocyte/macrophages cells (rabbit monoclonal, clone KP-1; Ventana, Tucson, AZ, USA) and CD3 antibody anti-T cells (rabbit monoclonal, clone 2GV6; Ventana). In addition, immunohistochemical reactions were performed in a small cohort of patients (15 female and 15 male) to study the expression of two interleukins (ILs) involved in the lymphocytes-related inflammatory response, IL-2 and IL-6. To this end, an anti-IL-2 rabbit monoclonal antibody (clone EPR2780; AbCam, Cambridge, UK) and an anti-IL-6 mouse polyclonal antibody (AbCam, Cambridge, UK) have been used.

Histopathologic examination was performed, utilizing the definitions reported above, by two blinded pathologists (A.M., F.S.). Inter-observer reliability was > 98 %.

### Risk factors definition

Clinical records were reviewed for all cases to determine risk factors profile.

According to AHA Scientific Statements [1] the presence of any 3 of 5 following conditions constitutes a diagnosis of metabolic syndrome: (a) elevated waist circumference (abdominal obesity) > 94 cm in men and > 80 cm in women; (b) hypertriglyceridemia: patients with serum triglycerides levels ≥ 150 mg/dL (> 1.70 mmol/L); (c) reduced HDL-C, < 40 mg/dL in men or < 50 mg/dL in women; (d) elevated blood pressure, systolic ≥ 130 and/or diastolic ≥ 85 mm Hg; (e) elevated fasting glucose ≥ 100 mg/dL.

The presence of hypertension was also evaluated according to the American College of Cardiology/American Heart Association (ACC/AHA) [27], the European Society of Cardiology (ESC) and European Society of Hypertension (ESH) Guidelines [28].

The other following major risk factors were considered: (a) hypercholesterolemia: patients with total cholesterol level > 200 mg/dL (> 5.18 mmol/L); (b) diabetes mellitus: patients with fasting blood glucose > 126 mg/dL and/or following oral treatment or insulin therapy; (c) patients with tobacco dependence were categorized as: smokers, if the consumption was more than 10 cigarettes/day, while who had stopped smoking for > 5 years was considered as non-smokers.

In order to evaluate levels of atherogenic cholesterol, low-density lipoprotein cholesterol (LDL-C) was calculated by the Friedewald equation [29] as follows: $LDL-C = cholesterol - (HDL-C + (triglycerides/5))$. A value of LDL-C of > 100 mg/dL was used as cut-off between high and low levels.

### Statistical analysis

Data were analyzed using SPSS version 16.0 (SPSS Inc, Chicago, Ill) software. Continuous variables were expressed as the mean ± SD or ± SE. The Shapiro-Wilk test was used to statistically assess the normal distribution of the data. Comparisons between continuous variables were performed using the independent Student *t*-test or the Wilcoxon rank sum test. Categorical data were analysed using the chi-square test or the Fisher exact test.

Multivariate analysis using stepwise logistic regression (using the “enter” method for variable selection) was utilized to identify independent risk factors which significantly correlate with the presence of plaque destabilization. The following variables were included: age, gender, hypertension, diabetes, smoking habit, hypercholesterolemia, low HDL and hypertriglyceridemia, abdominal obesity, metabolic syndrome, use of statins and anti-hypertensive drugs. Multivariate analysis was performed in 3 models: (1) using the definition of hypertension according to the ACC/AHA, (2) using the pressure cut-off of ESC/ESH Guidelines; (3) when the metabolic syndrome was considered in the multivariate analysis, hypertension, diabetes, hypertriglyceridemia, low HDL, and abdominal obesity were excluded. Similarly, when LDL-C was included in multivariate analysis, hypercholesterolemia, hypertriglyceridemia and low HDL were excluded. The odds ratio of an unstable plaque for the different risk factors was evaluated by logistic regression using the value of EXP (B), where B represents the logistic coefficient.

Moreover, multivariate logistic regression was also used in order to evaluate the effect of each component of metabolic syndrome in plaque destabilization. In this analysis only patients with the metabolic syndrome were considered.

A 2-tailed *p* value < 0.05 was considered statistically significant.

## Results

### Baseline data

Baseline data of patients are reported in Table 1.

**Table 1:** Baseline characteristics of patients

	N(%) or mean (SD)
Total	N = 186
Age	72.6 (8.6)
Gender	
Male	131 (70.4 %)
Female	55 (29.6 %)
Cerebrovascular disease	
Symptomatic patients	74 (39.8 %)
Ipsilateral major stroke	44 (23.7 %)
TIA	30 (16.1 %)
Asymptomatic patients	112 (60.2 %)
Risk factors	
Hypertension AHA [27]	162 (87.1 %)
Hypertension ESC [28]	112 (60.2 %)
Diabetes	80 (43.0 %)
Smoking habit	40 (21.5 %)
Hypercholesterolemia	29 (15.6 %)
Hypertriglyceridemia	70 (37.6 %)
Low-HDL	86 (46.2 %)
High LDL-C	71 (38.2 %)
IRC	56 (30.1 %)
Metabolic syndrome	85 (45.7 %)
Drugs	
Statins	128 (68.8 %)
Anti-hypertensive drugs	153 (82.3 %)
Associated vascular disease	
Acute cardiovascular disease	45 (24.2 %)
Previous myocardial infarction	37 (19.9 %)
Unstable angina	8 (4.3 %)
Peripheral arterial disease	61 (32.8 %)
Aortic aneurysm	11 (5.9 %)
Histological type of carotid plaque	
Stable plaques	104 (55.9 %)
Fibroatheromata	71 (38.2 %)
Fibrocalcific	33 (17.7 %)
Unstable plaques	82 (44.1 %)
Thrombotic plaque	48 (25.8 %)
With a thrombus in organization	16 (8.6 %)
TCFA	16 (8.6 %)
Calcified nodule	2 (1.1 %)

The mean age of 186 patients at time of surgical CEA was 72.6 ± 8.6 years, 131 (70.4 %) were male and 55 (29.6 %) were female. Seventy-two (38.7 %) patients were symptomatic (affected by ipsilateral major stroke or TIA), while 114 (61.3 %) were asymptomatic who underwent CEA for high grade carotid stenosis (> 60 %), assessed by echography or, in rare cases, by bilateral CT angiography.

A metabolic syndrome was observed in 85 out of 186 patients (45.7 %). All patients included in this study presented at least one risk factor. Among single risk factors, the hypertension was the most frequently observed when it was evaluated according to both the ACC/AHA (162 patients, 87.1 %) and to the ESC/ESH criteria (112 patients, 60.2 %). The low incidence of hypercholesterolemia (only 15.6 % of cases) may probably be explained by the 128 patients (68.8 %) who underwent statins treatments. Continuous treatment with aspirin (100 mg/die) was administered to patients with previous acute cardiovascular events (myocardial infarction, unstable angina) and with peripheral arterial disease during the pre-operative period Also, aspirin (100 mg/die) was administered to all patients during both the post-operative and follow-up periods. However, there were no statistical differences in the incidence of stable and unstable plaques by comparing patients treated with aspirin during the preoperative with untreated ones.

In 82 out of 186 patients (44.1 %) unstable plaques were found (Fig. 1). They consisted in 48 thrombotic plaques (all from symptomatic patients) associated with rupture of a thin fibrous cap rich in inflammatory cells, 16 TCFA (10 from symptomatic and 6 from asymptomatic patients) and 16 plaques with an organized acute thrombus (all symptomatic) characterized by a network of large, thin-walled vascular channels and a variable number of macrophagic cells loaded with hemosiderin within the area of an acute thrombus. In the remaining 2 unstable plaques, a calcified nodule was found protruding into the lumen covered by extremely thin fibrous cap. The remaining 104 carotids (55.9 % of cases) showed a stable plaque, characterized by a variable lipid-necrotic core containing extracellular lipid, cholesterol crystals and necrotic debris covered by a thick fibrous cap with few inflammatory cells (Fig. 1). In 33 plaques (both stable and unstable) a large calcification was observed.

As concern the immunohistochemical analysis, a significant increase in the number of both IL-2 and IL-6 positive inflammatory cells was observed in patients with instable plaques if compared to those with stable ones (IL-2 unstable 46.21 ± 3.1 cells/mm^2^ vs. stable 12.16 ± 2.331 cells/mm^2^, *p* = 0.01; IL-6 unstable 55.19 ± 4.22 cells/mm^2^ vs. stable 32.11 ± 3.09 cells/mm^2^, *p* = 0.01). Noteworthy, the number IL-6 positive inflammatory cells were significantly increased in unstable plaques of female patients as compared to unstable ones in male patients (female 71.13 ± 9.97 cells/mm^2^ vs. male 51.63 ± 5.99 cells/mm^2^, *p* = 0.02).

### Plaque instability and risk factors

The presence of unstable carotid plaques was not correlated with the finding of specific risk factors or to the use of statins and anti-hypertensive drugs, as demonstrated by the uni- and multivariate analysis (see Table 2). Only a significant correlation with the gender was observed, as males showed a higher incidence of unstable plaques than females (*p* = 0.01). In fact, unstable plaques were observed in 66 of the 131 (50.45 %) male patients undergoing CEA and only in 16 of the 55 (29.1 %) female patients.

**Table 2:** Plaque instability and risk factors

	Stable plaques (104 cases)	Unstable plaques (82 cases)	Odds ratio (95% CI)	P uni-variate analysis	P multi- variate analysis
Age (yrs ± SD)	72.5 ± 8.1	72.8 ± 9.3	0.99 (0.96–1.03)	0.80	0.87
Gender					
Male	65 (62.5%)	66 (80.5%)	0.41 (0.20–0.82)	0.008	0.01
Female	39 (37.5%)	16 (19.5%)			
Hypertension acc. AHA	90 (86.5%)	72 (87.8%)	1.47 (0.57–3.84)	0.80	0.42
Diabetes	48 (46.2%)	32 (39.0%)	0.70 (0.35–1.39)	0.33	0.31
Smoking habit	23 (22.1%)	17 (20.7%)	0.80 (0.36–1.73)	0.82	0.58
Hypercholesterolemia	17 (16.3%)	12 (14.6%)	0.65 (0.26–1.63)	0.75	0.35
Hypertriglyceridemia	35 (33.7%)	35 (42.7%)	1.80 (0.93–3.63)	0.21	0.10
Low-HDL	48 (46.2%)	38 (46.3%)	1.24 (0.63–2.45)	0.98	0.53
Abdominal obesity	20 (19.2%)	9 (11.0%)	0.47 (0.19–1-17)	0.12	0.11
Statins	74 (71.2%)	54 (65.9%)	0.75 (0.38–1.48)	0.44	0.41
Anti-hypertensive drugs	87 (83.7%)	66 (80.5%)	0.83 (0.36–1.91)	0.57	0.67
Hypertension acc. ESC	62 (59.6%)	50 (61.0%)	1.16 (0.60–2.24)	0.85	0.66
High LDL-C	38 (36.5%)	33 (40.2%)	1.17 (0.64–2.12)	0.61	0.30
Metabolic syndrome	49 (47.1%)	36 (43.9%)	0.91 (0.49–1.71)	0.66	0.77

In particular, the incidence of unstable plaques in patients with metabolic syndrome was 42.4 % (36 out of 85 cases), with no significant differences compared to that of stable plaques (*p* = 0.77).

When only the individual risk factors were considered in the statistical analysis, the greatest odds ratio for an unstable plaque was observed in patients with hypertriglyceridemia (1.80, 95 % CI 0.93–3.63) or hypertension (according to ACC/AHA) (1.47, 95% CI, 0.57–3.84). Considering the metabolic syndrome instead of hypertension, diabetes, hypertriglyceridemia, low HDL-C and abdominal obesity, a low odds ratio for plaque instability (0.91, 95% CI, 0.49–1.71) were found by multivariate analysis.

### Components of metabolic syndrome and plaque instability

Taking into account only the patients with metabolic syndrome, we evaluated the effect of single risk factors on plaque destabilization.

Patients with hypertriglyceridemia were those who presented a greater risk of destabilization of plaques (odds-ratio 1.56, 95% CI, 0.50–4.89). Since a significant inverse correlation between age and triglyceridemia values was observed (r = − 0.44, *p* = 0.001), the risk was calculated in two subgroups of patients with metabolic syndrome, with an age of ± 70 years old. Patients < 70 years old had an odds ratio of 1.55 (95% CI, 0.25–9.54), while in the elderly it was reduced to 1.00 (95% CI, 0.17–5.76). The risk was significantly increased in women with hypertriglyceridemia compared to males. In fact, the odds ratio was 1.28 (95% CI, 0.37–4.48) in the male subgroup and 3.01 (95% CI, 0.25–36.30) in the female one.

The other possible combinations of the metabolic syndrome components did not achieve a significant increase in risk.

## Discussion

The results obtained in our study show that MetS is not associated with a significant increase in the risk of carotid plaque destabilization, regardless of the analyzed risk factors. On the contrary, MetS showed a correlation with the presence of stable plaques according to the histological features released by the ACC/AHA [27]. Indeed, the incidence of unstable plaques in patients with MetS is rather low (43.9 %), when compared with that observed in the presence of other individual risk factors such as hypertension. However, the risk of carotid plaque destabilization significantly increases in the subgroup of female patients with hypertriglyceridemia, showing an odds ratio of 3.01. According to the known pro-atherogenic properties of IL-6, especially for what concern its capability to recruit and stimulate macrophages [30], we also observed a significant increase of IL-6 positive cells in unstable plaque of female patients, confirming previous observations that showed an increase in IL-6 responses in post-menopausal women [31].

Carotid stenosis still remains the criterion of choice to undergo a preventive endarterectomy, especially in asymptomatic patients [19–22]. According to our previous studies, results of the present work showed that more than 50 % of asymptomatic patients undergoing surgery had a stable carotid plaque that evolves very slowly over time [14, 15, 32]. This problem is particularly important in those asymptomatic patients with unilateral carotid stenosis who have no other comorbidities that may increase the risk of an ischemic stroke. The prevention of atherosclerosis is based on the control of the major cardiovascular risk factors and each patient has many of them, whose interaction must be taken into consideration. The various risk factors probably promote the slow growth of the carotid plaque. However, there are conflicting data which lead to the hypothesis that they can significantly determine not only the plaque growth but also its rupture and thrombosis.

### High‐risk carotid plaque

In our study both carotid vulnerable plaques and those with an acute thrombus were considered at high-risk. According to literature, carotid plaques morphologically characterized by a thin fibrous cap, heavily infiltrated by macrophages, without plaque rupture were considered as vulnerable [33]. As above defined [33] such plaques are at increased risk of thrombosis. Therefore, they are commonly included in the unstable plaques group. This group also include plaques with acute luminal thrombosis arising from either capsule rupture or capsule erosion (loss of endothelial lining). Recently,the calcific nodules, consisting of an eruptive, dense, calcified mass protruding into the lumen with an irregular surface, have been included among the unstable plaques group. They represent the hallmark of an unstable plaque subtype devoid of inflammation, as frequently show a discontinuity of the thin fibrous cap associated with an overlying luminal thrombus. Healed plaques with an organizing thrombus and presence of immature microvessels are typically included in the unstable plaques group. The neovascularization is necessary for plaque development. In a previous work [34], we demonstrated that a more extensive plaque neovascularization is associated with features of plaque vulnerability and with clinically symptomatic disease. These microvessels are immature and fragile and thus prone to rupture and hemorrhage, thus promoting plaque instability. Similarly, endothelial cells in plaque neovessels express more adhesion molecules than those in the main arterial lumen, which support leukocyte recruitment. Neovascularization assessed by contrast-enhanced ultrasound imaging is associated with plaque echolucency, a well-accepted marker of high-risk lesions, and it does not correlate on the degree of stenosis. In our cases, the presence of immature microvessels was histologically always associated with the presence of thrombotic plaques or plaques with thrombosis in organization and represented a constant histological aspect of these lesions. On the contrary, stable fibroatheromatous or fibrocalcific plaques showed only the presence of rare immature microvessels.

### Carotid plaque destabilization and risk factors

The results of the multivariate analysis reported in our study showed that none of the considered risk factors were independently associated with an increased risk of destabilization of the carotid plaque (Table 2). In particular, unstable carotid plaques were more frequent in hypertensive patients but, according to both the AHA [27] and ESC Guidelines [28], the assessed incidence of hypertension was equally high even in patients with stable plaques (*p* = 0.50 and 0.74, respectively). About 40 % of patients with unstable plaque were also affected by the MetS itself; nevertheless, a similar incidence was observed in those with stable plaque (*p* = 0.62). However, our results do not contradict some clinical meta-analyses, that propose the MetS as a relevant risk of developing cerebrovascular disease [5, 6, 35]. Among them, *Mottillo et al.* performed a large clinical meta-analysis by investigating data of 87 studies, comprising 951.083 subjects [35]. The increased risk, as our results have demonstrated, is not due to MetS itself but it varies on the basis of the combination of MetS components present in each patient. Only few and contradictory studies have assessed all possible combinations of MetS components in order to determine which elements are most strongly associated with atherosclerosis [8, 36–38]. In particular, Golden et al. [8] found a strong independent association between selected groupings of MetS components and excess carotid intimal-medial thickness, suggesting a synergy for atherosclerosis risk beyond what would have been expected from a merely additive effects. The evaluation of carotid intimal-medial thickness represents only a subclinical assessment of atherosclerosis [8, 24, 25] and does not distinguish between a stable plaque and ones with risk of rupture and thrombosis. A precise risk evaluation of plaque rupture can only be performed with histological methods (as in the present study) or with high-definition imaging approaches.

The major component of MetS that significantly correlates with an increased risk of destabilization of the carotid plaque was the hypertriglyceridemia.

In our series, hypertriglyceridemia represents the major component of dyslipidemia associated with the progression of atherosclerotic carotid plaque (Table 2). In fact, it should be emphasized that in our cases including patients undergoing endarterectomy (and therefore symptomatic or asymptomatic potentially at risk of acute cerebrovascular syndrome), the incidence of hypercholesterolemia (15.6 % of cases) and high LDL-C (38.2 % of cases) was very low. This can be explained by the fact that 128 out of 186 patients (68.8 %) were taking statins which, as it is well known, have a lowering effect on both LDL-C levels and anti-inflammatory effects.

### Carotid plaque destabilization, metabolic syndrome and triglycerides

Recent studies have long considered that elevated triglyceride levels increase the risk of cardiovascular disease that persists despite statin treatment [2, 39, 40]. Among patients with normal value of LDL-C, elevation of LDL-triglyceride significantly predicts the occurrence of major cardiovascular events, especially in diabetic or pre-diabetic patients [41, 42].

The PROVE-IT TIMI (Pravastatin or Atorvastatin Evaluation and Infection Therapy-Thrombolysis In Myocardial Infarction) trial further demonstrated that normal or low TG levels are associated with reduced cardiovascular event [43]. Patients who received statin treatment after hospitalization for acute coronary syndrome had a lower risk of further coronary heart disease events if triglyceride levels were < 150 mg/dL [43]. These results were confirmed by the recent REDUCE-IT (Reduction of Cardiovascular Events with Icosapent Ethyl–Intervention) clinical trial carried out on 8170 patients under statin therapy, with elevated fasting triglyceride levels and normal LDL-C levels [44]. This trial demonstrated a significant reduction in ischemic events in subjects taking Icosapent Ethyl (a molecule which decreases triglyceride levels without modifying LDL-C) as compared to subjects taking placebo, thus demonstrating how the reduction of triglyceride levels is associated with a lower risk of cardiovascular events among subjects with normal LDL-C levels [44]. Despite the normalization of LDL-C levels for the intake of statins, our results confirm that also in carotid district the increase in triglycerides is associated with an increase in the destabilization of atherosclerotic plaque, providing an evident morphological basis.

Hypertriglyceridemia could favor the destabilization of the carotid plaques by increasing local inflammation, damaging the endothelium, and stimulating the expression of cellular adhesion molecules [45, 46]. High levels of triglycerides are also associated with increased remnant lipoprotein particles that induce the expression of pro-inflammatory cytokines (TNF-α, IL-6, VCAM1, etc.) which have a cytotoxic effect on the endothelium [47].

The observation that the risk of plaque instability is higher in female patients with metabolic syndrome and hypertriglyceridemia, than in those without hypertriglyceridemia, confirms the conclusions of a recent meta-analysis by Li et al. [6]. The study reported that patients with MetS had a significantly higher risk of incident stroke, as compared to those without MetS, and that this effect was most remarkable among females (RR, 1.83 [95 % CI, 1.31–2.56]) compared with males (RR, 1.47 [95 % CI, 1.22–1.78]). Our histological data show that hypertriglyceridemia is a risk factor for carotid atherosclerosis especially in elderly women further confirming some observations based on other methodologies. La Fata et al. [48] found that, in a sample of post-menopausal women aged > 60 years, high concentrations of triglyceride-rich-lipoproteins (TRLPs) are independently associated with the risk of carotid atherosclerosis, evaluated by intima-media thickness ultrasound measurement. Moreover, Ahmad et al. [49] showed that adiposity might increase the genetic influences on hypertriglyceridemia among women and in particular these effects appear stronger for very-large-TRLPs subfraction. In our series, however, the presence of obesity does not increase the risk of plaque instability in women with hypertriglyceridemia and MetS.

Sex-difference in cardiovascular disease is now widely accepted [50]. Most likely, sex hormones play an atherosclerotic protective role in women, due to their action on endothelial function and lipid homeostasis. Estrogens might have plaque stabilization properties and effects on plaque inflammatory status [51, 52]. The effects of estrogens are probably age-dependent, reducing inflammation in younger age, while an opposite pro-inflammatory effect could be observed in older women, as those analyzed in the present study [53, 54].

### Study limitation

A possible limitation of this histologic study is that the analysis was performed by comparing the histological aspects of the carotid plaques rather than the presence of cerebrovascular symptoms. However, only the histological examination of plaques allows us to evaluate as objectively as possible whether they were stable, unstable, at risk of rupture, or already thrombotic. Furthermore, since one of the main targets for stroke prevention is the identification of plaque at risk, especially by imaging methods, we believe it is more useful to compare patients based on the histological aspect of the plaque rather than on symptoms. Finally, it should be noted that in our series all the 74 symptomatic patients had unstable plaques (while only 6 vulnerable unstable plaques were observed in asymptomatic patients). Therefore, it was not possible to perform separately the multivariate statistical analysis (stable vs. unstable plaques) in the group of symptomatic and asymptomatic patients.

## Conclusions

Data here reported suggest that a better understanding of how each individual component of MetS influences the stroke risk correlated to carotid plaque destabilization, rupture, and thrombosis, may help to stratified patients according to the real risk of acute cerebrovascular diseases. Moreover, our results support the hypothesis that the control of hypertriglyceridemia should be a key point on prevention of the destabilization of atherosclerotic carotid plaque, especially in post-menopausal female patients. However, data on sex-difference are still limited, and further studies are recommended to better define which differences may have implications for clinical prevention and management of acute complications of carotid artery disease.

## Data Availability

The data that support the findings of this study are available from the corresponding author upon reasonable request.

## Abbreviations

MetS: Metabolic syndrome
HDL-C: High-Density Lipoprotein cholesterol
IMT: Intimal-Medial Thickness
TIA: Transient ischemic attack
CEA: Carotid endarterectomy
TCFA: Thin-cap fibro-atheroma
LDL-C: Low-density lipoprotein cholesterol
DM: Diabetes mellitus
Pre-DM: Pre-diabetes mellitus
TRLPs: Triglyceride-rich-lipoproteins

## References

[1] Alberti KG, Eckel RH, Grundy SM, Zimmet PZ, Cleeman JI, Donato KA, Fruchart JC, James WP, Loria CM, Smith SC (2009). Harmonizing the metabolic syndrome: a joint interim statement of the International Diabetes Federation Task Force on Epidemiology and Prevention; National Heart, Lung, and Blood Institute; American Heart Association; World Heart Federation; International Atherosclerosis Society; and International Association for the Study of Obesity. Circulation.

[2] Benjamin EJ, Muntner P, Alonso A, Bittencourt MS, Callaway CW, Carson AP, Chamberlain AM, Chang AR, Cheng S, Das SR (2019). Heart disease and stroke statistics-2019 update: a report from the american heart association. Circulation.

[3] Li X, Fang F, Fu X, Lin H, Gao Q (2017). Is metabolic syndrome associated with the risk of recurrent stroke: a meta-analysis of Cohort Studies. J Stroke Cerebrovasc Dis.

[4] Roever L, Resende ES, Diniz ALD, Penha-Silva N, O’Connell JL, Gomes PFS, Zanetti HR, Roerver-Borges AS, Veloso FC, Fidale TM (2018). Metabolic syndrome and risk of stroke: protocol for an update systematic review and meta-analysis. Medicine.

[5] Gami AS, Witt BJ, Howard DE, Erwin PJ, Gami LA, Somers VK, Montori VM (2007). Metabolic syndrome and risk of incident cardiovascular events and death: a systematic review and meta-analysis of longitudinal studies. J Am Coll Cardiol.

[6] Li X, Lin H, Fu X, Lin W, Li M, Zeng X, Gao Q (2017). Metabolic syndrome and stroke: a meta-analysis of prospective cohort studies. J Clin Neurosci.

[7] Kernan WN, Inzucchi SE, Viscoli CM, Brass LM, Bravata DM, Shulman GI, McVeety JC, Horwitz RI (2003). Impaired insulin sensitivity among nondiabetic patients with a recent TIA or ischemic stroke. Neurology.

[8] Golden SH, Folsom AR, Coresh J, Sharrett AR, Szklo M, Brancati F (2002). Risk factor groupings related to insulin resistance and their synergistic effects on subclinical atherosclerosis: the atherosclerosis risk in communities study. Diabetes.

[9] Kernan WN, Viscoli CM, Furie KL, Young LH, Inzucchi SE, Gorman M, Guarino PD, Lovejoy AM, Peduzzi PN, Conwit R (2016). Pioglitazone after ischemic stroke or transient ischemic attack. N Engl J Med.

[10] Cardellini M, Rizza S, Casagrande V, Cardolini I, Ballanti M, Davato F, Porzio O, Canale MP, Legramante JM, Mavilio M (2019). Soluble ST2 is a biomarker for cardiovascular mortality related to abnormal glucose metabolism in high-risk subjects. Acta Diabetol.

[11] Botvin Moshe C, Haratz S, Ravona-Springer R, Heymann A, Hung-Mo L, Schnaider Beeri M, Tanne D (2020). Long-term trajectories of BMI predict carotid stiffness and plaque volume in type 2 diabetes older adults: a cohort study. Cardiovasc Diabetol.

[12] Hansson GK, Libby P (2006). The immune response in atherosclerosis: a double-edged sword. Nat Rev Immunol.

[13] Finn AV, Nakano M, Narula J, Kolodgie FD, Virmani R (2010). Concept of vulnerable/unstable plaque. Arterioscler Thromb Vasc Biol.

[14] Spagnoli LG, Mauriello A, Sangiorgi G, Fratoni S, Bonanno E, Schwartz RS, Piepgras DG, Pistolese R, Ippoliti A, Holmes DR (2004). Extracranial thrombotically active carotid plaque as a risk factor for ischemic stroke. JAMA.

[15] Mauriello A, Servadei F, Sangiorgi G, Anemona L, Giacobbi E, Liotti D, Spagnoli LG (2011). Asymptomatic carotid plaque rupture with unexpected thrombosis over a non-canonical vulnerable lesion. Atherosclerosis.

[16] Mauriello A, Sangiorgi GM, Virmani R, Trimarchi S, Holmes DR, Kolodgie FD, Piepgras DG, Piperno G, Liotti D, Narula J (2010). A pathobiologic link between risk factors profile and morphological markers of carotid instability. Atherosclerosis.

[17] Scimeca M, Anemona L, Granaglia A, Bonfiglio R, Urbano N, Toschi N, Santeusanio G, Schiaroli S, Mauriello S, Tancredi V (2019). Plaque calcification is driven by different mechanisms of mineralization associated with specific cardiovascular risk factors. Nutr Metab Cardiovasc Dis.

[18] Cardellini M, Rovella V, Scimeca M, Anemona L, Bischetti S, Casella S, Saggini A, Bonanno E, Ballanti M, Davato F (2019). Chronic kidney disease is linked to carotid nodular calcification, an unstable plaque Not correlated to inflammation. Aging Dis.

[19] Kernan WN, Ovbiagele B, Black HR, Bravata DM, Chimowitz MI, Ezekowitz MD, Fang MC, Fisher M, Furie KL, Heck DV (2014). Guidelines for the prevention of stroke in patients with stroke and transient ischemic attack: a guideline for healthcare professionals from the American Heart Association/American Stroke Association. Stroke.

[20] Eckstein HH (2018). European society for vascular surgery guidelines on the management of atherosclerotic carotid and vertebral artery disease. Eur J Vasc Endovasc Surg.

[21] Halliday A, Harrison M, Hayter E, Kong X, Mansfield A, Marro J, Pan H, Peto R, Potter J, Rahimi K (2010). 10-year stroke prevention after successful carotid endarterectomy for asymptomatic stenosis (ACST-1): a multicentre randomised trial. Lancet.

[22] Tsivgoulis G, Safouris A, Kim DE, Alexandrov AV (2018). Recent advances in primary and secondary prevention of atherosclerotic stroke. J Stroke.

[23] Guembe MJ, Fernandez-Lazaro CI, Sayon-Orea C, Toledo E, Moreno-Iribas C (2020). Risk for cardiovascular disease associated with metabolic syndrome and its components: a 13-year prospective study in the RIVANA cohort. Cardiovasc Diabetol.

[24] Kumar P, Sharma R, Misra S, Kumar A, Nath M, Nair P, Vibha D, Srivastava AK, Prasad K (2020). CIMT as a risk factor for stroke subtype: a systematic review. Eur J Clin Invest.

[25] Willeit P, Tschiderer L, Allara E, Reuber K, Seekircher L, Gao L, Liao X, Lonn E, Gerstein HC, Yusuf S (2020). Carotid intima-media thickness progression as surrogate marker for cardiovascular risk: Meta-analysis of 119 clinical trials involving 100 667 patients. Circulation.

[26] Virmani R, Kolodgie FD, Burke AP, Farb A, Schwartz SM (2000). Lessons from sudden coronary death: a comprehensive morphological classification scheme for atherosclerotic lesions. Arterioscler Thromb Vasc Biol.

[27] Whelton PK, Carey RM, Aronow WS, Casey DE, Collins KJ, Dennison Himmelfarb C, DePalma SM, Gidding S, Jamerson KA, Jones DW (2018). 2017 ACC/AHA/AAPA/ABC/ACPM/AGS/APhA/ASH/ASPC/NMA/PCNA guideline for the prevention, detection, evaluation, and management of high blood pressure in adults: executive summary: a report of the American College of Cardiology/American Heart Association Task Force on Clinical Practice Guidelines. J Am Coll Cardiol.

[28] Williams B, Mancia G, Spiering W, Agabiti Rosei E, Azizi M, Burnier M, Clement DL, Coca A, de Simone G, Dominiczak A (2018). 2018 ESC/ESH Guidelines for the management of arterial hypertension: the Task Force for the management of arterial hypertension of the European Society of Cardiology and the European Society of Hypertension: the Task Force for the management of arterial hypertension of the European Society of Cardiology and the European Society of Hypertension. J Hypertens.

[29] Friedewald WT, Levy RI, Fredrickson DS (1972). Estimation of the concentration of low-density lipoprotein cholesterol in plasma, without use of the preparative ultracentrifuge. Clin Chem.

[30] Eltoft A, Arntzen KA, Wilsgaard T, Mathiesen EB, Johnsen SH (2018). Interleukin-6 is an independent predictor of progressive atherosclerosis in the carotid artery: the Tromso Study. Atherosclerosis.

[31] Endrighi R, Hamer M, Steptoe A (2016). Post-menopausal women exhibit greater interleukin-6 responses to mental stress than older men. Ann Behav Med.

[32] Mauriello A, Sangiorgi G, Virmani R, Servadei F, Trimarchi S, Holmes DR, Kolodgie F, Biondi Zoccai G, Leuzzi C, Spagnoli LG (2012). Evidence of a topographical link between unstable carotid plaques and luminal stenosis: can we better stratify asymptomatic patients with significant plaque burden?. Int J Cardiol.

[33] Muller JE, Tofler GH, Stone PH (1989). Circadian variation and triggers of onset of acute cardiovascular disease. Circulation.

[34] Coli S, Magnoni M, Sangiorgi G, Marrocco-Trischitta MM, Melisurgo G, Mauriello A, Spagnoli L, Chiesa R, Cianflone D, Maseri A (2008). Contrast-enhanced ultrasound imaging of intraplaque neovascularization in carotid arteries: correlation with histology and plaque echogenicity. J Am Coll Cardiol.

[35] Mottillo S, Filion KB, Genest J, Joseph L, Pilote L, Poirier P, Rinfret S, Schiffrin EL, Eisenberg MJ (2010). The metabolic syndrome and cardiovascular risk a systematic review and meta-analysis. J Am Coll Cardiol.

[36] Haffner SM, D’Agostino R, Mykkanen L, Tracy R, Howard B, Rewers M, Selby J, Savage PJ, Saad MF (1999). Insulin sensitivity in subjects with type 2 diabetes. Relationship to cardiovascular risk factors: the Insulin Resistance Atherosclerosis Study. Diabetes Care.

[37] Sheu WH, Jeng CY, Young MS, Le WJ, Chen YT (2000). Coronary artery disease risk predicted by insulin resistance, plasma lipids, and hypertension in people without diabetes. Am J Med Sci.

[38] Rovella V, Anemona L, Cardellini M, Scimeca M, Saggini A, Santeusanio G, Bonanno E, Montanaro M, Legramante IM, Ippoliti A (2018). The role of obesity in carotid plaque instability: interaction with age, gender, and cardiovascular risk factors. Cardiovasc Diabetol.

[39] Toth PP, Shah PK, Lepor NE (2020). Targeting hypertriglyceridemia to mitigate cardiovascular risk: a review. Am J Prev Cardiol.

[40] Schwartz GG, Abt M, Bao W, DeMicco D, Kallend D, Miller M, Mundl H, Olsson AG (2015). Fasting triglycerides predict recurrent ischemic events in patients with acute coronary syndrome treated with statins. J Am Coll Cardiol.

[41] Reiner Z (2017). Hypertriglyceridaemia and risk of coronary artery disease. Nat Rev Cardiol.

[42] Jin JL, Zhang HW, Cao YX, Liu HH, Hua Q, Li YF, Zhang Y, Guo YL, Wu NQ, Zhu CG (2020). Long-term prognostic utility of low-density lipoprotein (LDL) triglyceride in real-world patients with coronary artery disease and diabetes or prediabetes. Cardiovasc Diabetol.

[43] Miller M, Cannon CP, Murphy SA, Qin J, Ray KK, Braunwald E (2008). Impact of triglyceride levels beyond low-density lipoprotein cholesterol after acute coronary syndrome in the PROVE IT-TIMI 22 trial. J Am Coll Cardiol.

[44] Bhatt DL, Steg PG, Miller M, Brinton EA, Jacobson TA, Ketchum SB, Doyle RT, Juliano RA, Jiao L, Granowitz C (2019). Cardiovascular risk reduction with Icosapent ethyl for hypertriglyceridemia. N Engl J Med.

[45] Marz W, Scharnagl H, Winkler K, Tiran A, Nauck M, Boehm BO, Winkelmann BR (2004). Low-density lipoprotein triglycerides associated with low-grade systemic inflammation, adhesion molecules, and angiographic coronary artery disease: The Ludwigshafen Risk and Cardiovascular Health study. Circulation.

[46] Nordestgaard BG (2016). Triglyceride-rich lipoproteins and atherosclerotic cardiovascular disease: New insights from epidemiology, genetics, and biology. Circ Res.

[47] Doi H, Kugiyama K, Oka H, Sugiyama S, Ogata N, Koide SI, Nakamura SI, Yasue H (2000). Remnant lipoproteins induce proatherothrombogenic molecules in endothelial cells through a redox-sensitive mechanism. Circulation.

[48] La Fata E, Gentile M, Iannuzzi A, Covetti G, D'Elia L, Iannuzzo G, Panico S, Rubba P (2017). Association between Triglyceride-Rich-Lipoprotein subfractions and carotid atherosclerosis in post-menopausal women. Nutr Metab Cardiovasc Dis.

[49] Ahmad S, Mora S, Franks PW, Orho-Melander M, Ridker PM, Hu FB, Chasman DI (2018). Adiposity and genetic factors in relation to triglycerides and triglyceride-rich lipoproteins in the Women’s Genome Health Study. Clin Chem.

[50] Sangiorgi G, Roversi S, Biondi Zoccai G, Modena MG, Servadei F, Ippoliti A, Mauriello A (2013). Sex-related differences in carotid plaque features and inflammation. J Vasc Surg.

[51] Burke AP, Farb A, Malcom G, Virmani R (2001). Effect of menopause on plaque morphologic characteristics in coronary atherosclerosis. Am Heart J.

[52] Iemolo F, Martiniuk A, Steinman DA, Spence JD (2004). Sex differences in carotid plaque and stenosis. Stroke.

[53] Manson JE, Hsia J, Johnson KC, Rossouw JE, Assaf AR, Lasser NL, Trevisan M, Black HR, Heckbert SR, Detrano R (2003). Estrogen plus progestin and the risk of coronary heart disease. N Engl J Med.

[54] Xing D, Nozell S, Chen YF, Hage F, Oparil S (2009). Estrogen and mechanisms of vascular protection. Arterioscler Thromb Vasc Biol.

